# Low frequency of parental mosaicism in *de novo COL4A5* mutations in X‐linked Alport syndrome

**DOI:** 10.1002/mgg3.1452

**Published:** 2020-08-18

**Authors:** Ole Magnus Bjorgaas Helle, Torkild Høieggen Pedersen, Lilian Bomme Ousager, Mads Thomassen, Jens Michael Hertz

**Affiliations:** ^1^ Department of Clinical Genetics Odense University Hospital Odense Denmark; ^2^ Department of Clinical Research University of Southern Denmark Odense Denmark

**Keywords:** Alport syndrome, COL4A5, de novo mutation, mosaicism

## Abstract

**Background:**

Alport syndrome is a progressive hereditary kidney disease clinically presenting with haematuria, proteinuria, and early onset end‐stage renal disease, and often accompanied by hearing loss and ocular abnormalities. The inheritance is X‐linked in the majority of families and caused by sequence variants in the *COL4A5* gene encoding the α5‐chain of type‐IV collagen. The proportion of *de novo COL4A5* sequence variants in X‐linked Alport syndrome has been reported between 12 and 15% in previous studies.

**Methods:**

In the present study we have systematically investigated the mosaic status of asymptomatic parents of six patients with X‐linked Alport syndrome using next‐generation sequencing of DNA extracted from different tissues. The deleterious *COL4A5* sequence variants in these patients were previously assumed to be *de novo*, based on Sanger sequencing of the parents.

**Results:**

A low‐grade (1%) parental mosaicism was detected in only one out of six families (17%). In addition, in one out of six families (17%), we found that the mutational event probably occurred postzygotic.

**Conclusion:**

These findings highlight the importance of testing for mosaicism in unaffected parents of patients with sequence variants considered to be *de novo*, as it may have implications for the recurrence risk and thereby for the genetic counseling of the family.

## Graphical abstract

A low‐grade parental mosaicism was detected in one out of six patients with a de novo COL4A5 mutation in patients with X‐linked Alport syndrome.

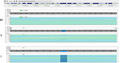

## 1 INTRODUCTION

Alport syndrome (AS) is a progressive hereditary kidney disease clinically presenting with haematuria, proteinuria, and early onset end‐stage renal disease (ESRD). It is often accompanied by high tone hearing loss and ocular abnormalities (Kruegel, Rubel, & Gross, 2013). The frequency of AS in the general population is unknown but the most commonly cited estimate of the prevalence is 1:5000, from a North American study (Hasstedt & Atkin, 1983). Two studies from the Nordic countries found an incidence of 1:17,000 in live births in southern Sweden (Persson, Hertz, Wieslander, & Segelmark, 2005) and 1:53,000 in Finland (Pajari, Kääriäinen, Muhonen, & Koskimies, 1996). The divergence in prevalence and incidence could be explained by variation in the clinical presentation in patients with AS, especially in female patients (Pajari et al., 1996).

The X‐linked form of AS (OMIM:301,050) constitutes about 85% of the cases, and is caused by sequence variants in the *COL4A5* gene (OMIM:303,630), located at Xq22.3, and encoding the α5‐chain of type‐IV collagen. The autosomal recessive form of AS (OMIM:203,780), that constitutes about 15% of the cases, and a rare autosomal dominant form of AS (OMIM:104,200), are caused by sequence variants in either *COL4A3* (OMIM:120,070) or *COL4A4* (OMIM:120,131), located at 2q36.3, and encoding the α3‐ and α4‐chains of type‐IV collagen, respectively. The sequence variant in either of these genes interferes with the formation of the mature α3α4α5 triple helix in type‐IV collagen, which is an integral part of the glomerular basement membrane.

The proportion of *de novo COL4A5* sequence variants in X‐linked AS has been reported to be between 12 and 15% in previous studies (Inoue et al., 1999; Jais et al., 2003). In apparently *de novo* cases, parental mosaicism can be detected in some instances and somatic mosaicism in the index case in others. Mosaicism is defined by the presence of two or more genotypically different populations of cells within the same individual, due to a mutation occurring at any point in the development of the individual (Biesecker & Spinner, 2013; Lupski, 2013). Depending on the timing of the occurrence of the mutation, the mosaicism may affect only somatic cells (somatic mosaicism), only germline cells (gonadal mosaicism) or both (gonosomal mosaicism), the latter being the case if the mutation arises within the first 15 cell divisions (Campbell et al., 2014). The timing of the mutation may also influence the grade of mosaicism. A mutation occurring in the early stages of development will potentially affect more cells, although selection may affect the grade of mosaicism at a later stage (Biesecker & Spinner, 2013).

A low‐grade mosaicism will not necessarily be detected by Sanger sequencing. Next‐generation sequencing (NGS) has the ability to test this by detecting the same nucleotide position multiple times, and has shown to be more sensitive than Sanger sequencing in detecting low‐grade mosaicisms (<20%) (Acuna‐Hidalgo et al., 2005; Beicht et al., 2013).

The aim of the present study was to analyze for parental mosaicism by deep sequencing using NGS of different tissues in parents of patients with X‐linked AS, caused by what was assumed to be a *de novo COL4A5* sequence variant by Sanger sequencing. As mosaicism in either of the parents increases the risk of having a second child with the same disorder, our results could have an impact on the recurrence risk, and thereby for the genetic counseling of parents of these children with X‐linked AS.

## 2 SUBJECTS AND METHODS

### 2.1 Ethical compliance

The Regional Committees on Health Research Ethics for Southern Denmark approved the study (Project number S‐20150115). All participants gave both written and oral informed consent. Five of the parents from which previously stored samples were available for study, were deceased at the time of investigation: both parents in family 2, the father in family 3, and the mother in families 4 and 5. The closest living relative gave consent for deceased individuals.

### 2.2 Patients and relatives

Six cases, all females, from six different families, previously referred to our laboratory for *COL4A5* (NM_000495.4) mutation analysis during the last more than 25 years, and 11 parents to the index cases, were included in the study. Clinical features of the index cases are summarized in Table 1. DNA from blood was available for study from all parents, except from the mother in family 3, who was deceased at the time of the investigation. Both parents in family 2, the father in family 3, and the mother in families 4 and 5 were also deceased at the time of investigation, but DNA from blood was stored from previous investigations. DNA from buccal cells was available from 5/6 (86%) of the index cases, and from 6/11 (55%) of the parents. DNA from urine was available from 4/6 (67%) of the index cases, and 3/11 (33%) of the parents. Urine samples were actually available from another four participants, but they contained inadequate amounts of DNA for NGS analysis. DNA from sperm cells was only available from the father in family 6.

**Table 1:** Clinical features and family history of the six index cases and their *COL4A5* (NM_000495.4) sequence variants

Family	Sex of proband	Age of proband (years)	Affected relatives	Hematuria/proteinuria	*COL4A5* sequence variant (heterozygous) Exon Nucleotide change Protein change			Previously published (reference)
1	Female	43	One daughter	+/+	10	c.594_597dupACCA	p.(Pro200fsX17)	No
2	Female	69	Two sons and one daughter	+/+	20	c.1219C=T	p.(Gln407X)	Yes (Hertz et al., 2001; Martin et al., 1998)
3	Female	77	One son	+/−	24	c.1718G=A	p.(Gly573Asp)	Yes (Hertz et al., 2001; Martin et al., 1998)
4	Female	61	Three sons	+/−	25	c.1856C=T	p.(Pro619Leu)	Yes (Hertz et al., 2001; Palenzuela et al., 2002)
5	Female	50	One son and one daughter	+/−	27	c.2057delC	p.(Pro686fsX50)	Yes (Tazon‐Vega et al., 2007)
6	Female	16	None	+/−	32	c.2686G=T	p.(Gly896Cys)	Yes (Daga et al., 2018)

**Figure 1:**
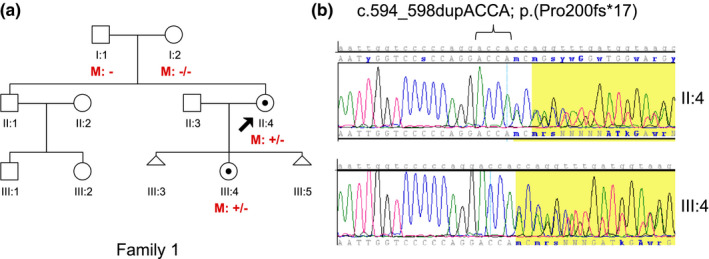
(a) Pedigree of family 1. The novel *COL4A5* (NM_000495.4) sequence variant: c.594_598dupACCA is present in heterozygous form in the index case (II:4, marked with an arrow), and her daughter (III:4), but not in the parents (I:1 and I:2). (b) Sanger sequencing of *COL4A5* exon 10 of the index case (II:4) and her daughter (III:4)

All six index cases were previously diagnosed with X‐linked AS and found to have a pathogenic *COL4A5* sequence variant that based on Sanger sequencing of samples from the parents seemed to be *de novo*. Five of the *COL4A5* sequence variants have previously been reported in the literature, and one is novel: a heterozygous frameshift mutation in exon 10, c.595_597dupACCA; p.(200fsX17), detected in the index case in family 1 (Table 1 and Figure 1). The parental origin of the X‐chromosome harboring the *COL4A5* sequence variant in three of the cases and the paternity have been determined by haplotype analysis using closely linked polymorphic markers (data not shown). The sequencing of the *COL4A3* and *COL4A4* genes was normal.

All index cases presented with micro‐ and/or episodes of macroscopic haematuria. Proteinuria had been detected in the index cases in families 1 and 2. A kidney biopsy obtained from the index case in families 1 and 6 showed characteristic GBM changes. None of the index cases have hearing loss or ocular abnormalities. All index cases, except for the index case in family 6, a 16‐years‐old female, have affected children, and all affected descendants are having the same *COL4A5* sequence variant as detected in the index case. All pedigrees are in accordance with X‐linked inheritance. None of the parents of the index cases have clinical features of AS.

### 2.3 Sample purification and DNA extraction

DNA for next‐generation sequencing was obtained from different sources depending on availability. DNA from EDTA‐stabilized peripheral blood lymphocytes was extracted using standard methods. DNA purification from buccal cells, urine samples, and sperm cells was performed using Maxwell 16 Buccal Swab LEV DNA purification kit AS 1295, Maxwell 16 Cell LEV DNA purification kit AS 1140, and Maxwell 16 Tissue DNA purification kit AS 1030, respectively. DNA extraction was performed on an AS 1000 Maxwell 16 platform (Promega, USA).

### 2.4 Library construction and next‐generation sequencing

Sample DNA was fragmented by ultrasound using Covaris S220 Adaptive Focused Acoustics (Covaris, USA) technology. Sample preparation was performed on a Beckman Coulter Biomek 4000 Laboratory Automation Workstation using SureSelect XT Library Prep Kit ILM. Adaptors from Sigma Aldrich were used. DNA Enrichment was performed with SureSelect Target enrichment Kit from Agilent; library capture was performed with a custom Oligo Capture Library from Agilent. Targeted paired‐end sequencing (2x100 base pairs) was performed on an Illumina HiSeq 1500 platform with a mean coverage at the areas of mutation of 82X‐1666X reads for the blood samples, 75X‐1228X reads for the buccal cell samples, 13X‐595X for urine samples, and 446X for the single sperm sample.

### 2.5 Data analysis

Reads were processed using the Burrows‐Wheeler Alignment tool (BWA‐MEM) v. 0.7.12, GATK Best Practice pipeline v.3.3‐0 was used for variant calling, and ANNOVAR for the annotation of variants.

As a cut off level for variant detection, we have used a Phred Quality Score of 30 (Q30), corresponding to an error rate of 1 in 1000, that is, a base call accuracy of 99.9%.

## 3 RESULTS

**Figure 2:**
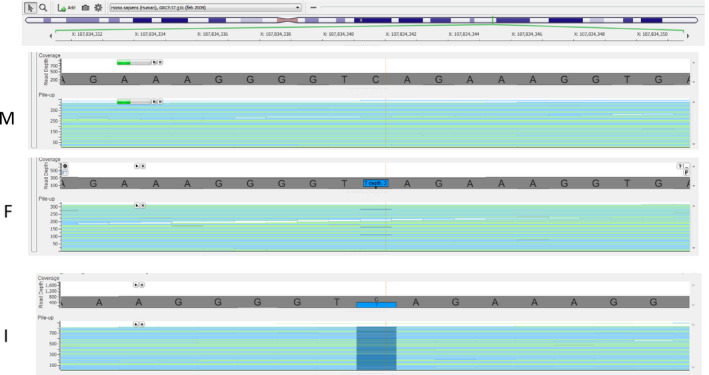
Family 2 with a parental *COL4A5* (NM_000495.4) mosaicism: c.1219C=T; p.(Gln407X), detected by next‐generation sequencing of DNA stored from blood samples. Reading depth information from the BAM files indicates that the variant nucleotide was present in approximately 50% of the reads in the index case (I), in 1% in the father (F), and not present in the mother (M). For details, see text and Table 2

The results of the analyses are summarized in Table 2. The mutated allele was detected in only one of the parents in one out of six families investigated by deep sequencing using NGS. The nonsense mutation, c.1219C=T; p.(Gln407X), detected in the index case in family 2, was detected in 3 out of 299 reads (1%) in her father and highly above the cut off level for variant detection (Figure 2). DNA from other tissues was not available from him, as he was deceased at the time of the investigation, and only DNA from blood was stored. The paternal origin of the mutation in this family is in accordance with the haplotype analysis of the family stating that the mutated allele in the index case was found to originate from the paternal X‐chromosome.

**Table 2:** No. reads and coverage in different tissues from the six index cases, all females, and their parents

Family		Tissue/cells analysed	Variant reads	Total reads	%	Mean coverage	Parental origin^a^
1	Index case	Blood:	159	373	43	286	Not determined
		Buccal:	219	644	34	520	
		Urine:	214	562	38	486	
	Mother	Blood:	0	424	0	290	
		Buccal:	0	1668	0	1228	
	Father	Blood:	0	163	0	125	
		Buccal:	0	130	0	121	
		Urine:	0	81	0	71	
2	Index case	Blood:	390	816	48	658	Paternal
		Buccal:	401	788	51	631	
		Urine:	7	22	32	17	
	Mother	Blood:	0	419	0	575	
	Father	Blood:	3	299	1	121	
3	Index case	Blood:	372	757	49	611	Paternal
		Buccal:	646	1270	51	1251	
	Father	Blood:	0	101	0	82	
4	Index case	Blood:	873	1751	50	1412	Paternal
		Buccal:	74	115	64	84	
		Urine:	20	38	53	38	
	Mother	Blood:	0	2050	0	1666	
	Father	Blood:	0	1198	0	1115	
		Buccal:	0	87	0	91	
		Urine:	0	188	0	197	
5	Index case	Blood:	138	303	46	288	Not determined
		Buccal:	194	386	50	399	
		Urine:	134	303	44	104	
	Mother	Blood:	0	231	0	230	
	Father	Blood:	0	534	0	499	
		Buccal:	0	79	0	75	
6	Index case	Blood:	1196	2418	49	1979	Not determined
	Mother	Blood:	0	573	0	492	
		Buccal:	0	133	0	110	
		Urine:	0	766	0	595	
	Father	Blood:	0	711	0	592	
		Buccal:	0	709	0	520	
		Sperm:	0	501	0	446	

^a^ The parental origin of X‐chromosome harboring the *COL4A5* (NM_000495.4) sequence variant has been determined in three of the families by haplotype analysis using polymorphic markers (Hertz, 2009).

The sequence variants detected in the other five index cases could not be detected in any of the investigated tissues from their respective parents. A parental mosaicism could, therefore, only be detected in one out of six (17%) of the families.

The novel frameshift mutation in the index case in family 1: c.594_597dupACCA, was detected by NGS in 43%, 34%, and 38% of the reads in DNA from blood, buccal cells, and urine, respectively, indicating a postzygotic mosaic state.

## 4 DISCUSSION

This study is the first to systematically investigate by NGS the mosaic status of asymptomatic parents of patients with X‐linked AS, in whom the disease‐causing *COL4A5* sequence variant was assumed to be *de novo*, based on Sanger sequencing.

We have detected low‐grade mosaicism (1%) for a previously reported *COL4A5* sequence variant, c.1219C=T; p.(Gln407X), in a blood sample from the father of the index case in family 2. As the father of the index case was deceased at the time of this investigation, no other tissues could be obtained for testing. The paternal origin of the mutation is in accordance with a previously performed segregation analysis in the same family. Using closely linked polymorphic markers it was demonstrated that the sequence variant in the index case in family 2 occurred on the paternal X‐chromosome (Hertz, 2009).

Analyses of the parents in the five other families were without evidence of mosaicism, suggesting that 1/6 (17%) of the probands, previously thought to have a *de novo* sequence variant, in fact, inherited the disease from an asymptomatic, mosaic parent.

In the present study, we obtained samples from different tissues derived from different germinal layers. By analyzing peripheral blood (mesoderm), buccal cells from oral mucosa (ectoderm), and urine cells (mesoderm/endoderm), we were able to investigate the percentage of mutant cells in all three germinal layers. This was performed for two reasons: First, the likelihood of finding mutated cell‐lines increases when investigating various tissues. Second, this allowed us to study possible variations between the different tissues. However, we were not able to obtain samples from all three different tissues from all the parents, mainly due to the fact that six of the parents were deceased at the time of this study, and only DNA from blood was stored from five of them.

The mean coverage varied considerably between the different samples, both between the same type of tissue from the different individuals and between the different types of tissue from the same individual (Table 2). This difference can to some extent be explained by differences in quality of the DNA and inadequate amount of DNA, especially from buccal cells and urine. Some of the DNA samples from blood have been stored for more than 25 years.

The proportion of *de novo* sequence variants in X‐linked AS has been reported between 12 and 15% in previous studies (Inoue et al., 1999; Jais et al., 2003). The parental origin of *de novo* mutations has not been investigated thoroughly for AS, but studies in other diseases have shown that *de novo* variants usually are of paternal origin (Kong et al., 2012). The higher number of divisions in spermatogenesis compared to oogenesis could explain this, as more divisions add to the risk of mutations occurring (Crow, 1997; Girard et al., 2001; Kong et al., 2012). However, for an X‐linked condition like AS, an affected male patient necessarily harbor the sequence variant on the X‐chromosome inherited from his mother. The parental origin in all three of the present female cases, in which an informative segregation analysis could be performed, showed that the sequence variant occurred on the paternal X‐chromosome.

Plant, Boye, Green, Vetrie, and Flinter (2000) identified *de novo* variants in 17.9% (5/28) of AS families by the screening of 25 mothers of affected male patients, and both parents of three affected females, using single‐stranded conformation polymorphism analysis and Sanger sequencing of DNA from blood. In addition, they found parental somatic mosaicism in three of the parents (10.7%), two of which have clinical features of AS. This is comparable to the 16.7% identified in the present study, although we have only included families with a normal result of Sanger sequencing of asymptomatic parents.

The frequency of the mutated allele may vary between different tissues (Beicht et al., 2013; Krol et al., 2008). Interestingly, the frequency of the mutated allele in samples from different tissues from the index case in family 1, were all below 50%. It was found to be 43% in blood, 34% in buccal cells, and 38% in urine sediments. The lower than expected percentage indicates high‐grade mosaicism in the index case, suggesting an early postzygotic mutational event. Somatic mosaicism has previously been reported in AS in several cases, and typically in patients with a milder phenotype (Krol et al., 2008; Fu et al., 2016; Okamoto, Nozu, Iijima, & Ariga, 2019). Beicht et al. (2013) analyzed the affected mother of a boy with X‐linked AS and found the mutated allele being present in 14%, 7%, 4%, and 7% in DNA from leukocytes, urine sediments, hair roots, and oral mucosa, respectively.

In conclusion, by NGS we found a low‐grade (1%) parental mosaicism in one out of six families (17%) in asymptomatic parents of children with X‐linked AS, in whom the disease‐causing *COL4A5* sequence variant was assumed to be *de novo*, based on Sanger sequencing.

In addition, in one out of six families (17%), we found that the mutational event in the index case probably occurred postzygotic.

Although based on a limited number of patients, these findings highlight the importance of testing for mosaicism in unaffected parents of cases with sequence variants considered to be *de novo*, as it may have implications for the recurrence risk and thereby for the genetic counseling of the family.

## CONFLICT OF INTEREST

The authors declare that they have no conflict of interest.

## AUTHOR CONTRIBUTIONS

Jens Michael Hertz conceptualized the study, Ole Magnus Bjorgaas Helle and Torkild Høieggen Pedersen collected the clinical information and laboratory data, and drafted the manuscript. Mads Thomassen, Lilian Bomme Ousager, and Jens Michael Hertz critically revised the manuscript. All authors contributed to the interpretation of data and gave approval for the final version of the manuscript.
